# Magnetically tunable broadband transmission through a single small aperture

**DOI:** 10.1038/srep12489

**Published:** 2015-07-22

**Authors:** Ke Bi, Wenjun Liu, Yunsheng Guo, Guoyan Dong, Ming Lei

**Affiliations:** 1 State Key Laboratory of Information Photonics and Optical Communications & School of Science, Beijing University of Posts and Telecommunications, Beijing 100876, China; 2 State Key Laboratory of New Ceramics and Fine Processing, School of Materials Science and Engineering, Tsinghua University, Beijing 100084, China; 3 College of Materials Science and Opto-Electronic Technology, University of Chinese Academy of Sciences, Beijing 100049, China

## Abstract

Extraordinary transmission through a small aperture is of great interest. However, it faces a limitation that most of approaches can not realize the tunable transmission property, which is not benefit for the miniaturization of the microwave system. Here, we demonstrate a magnetically tunable broadband transmission through a small aperture. By placing two ferrite rods symmetrically on both sides of a single small aperture, the strongly localized electromagnetic fields are effectively coupled to the two ferrite rods. Both the simulated and experimental results indicate that such structure not only realizes a nearly total transmission through a small aperture, but also obtains a magnetically tunable property. This work offers new opportunities for the miniaturization of the microwave system.

Extraordinary transmission through a small aperture, drilled in an optically thick metal film, has stimulated tremendous interests due to its potential applications in the fields of flat optics, nanolithography, solar cells, fluorescence, chemical sensors, spectral filters, optical trapping, and second harmonic generation^1^,^2^,^3^,^4^,^5^,^6^. Since Ebbesen *et al*.^7^ realized the extraordinary transmission from small aperture arrays that were milled in the thick metallic film, many configurations such as filling the hole with a material of high dielectric permittivity and placing artificially designed metamaterial covers in front of the aperture have been prepared to obtain efficient extraordinary transmission^8^,^9^,^10^,^11^,^12^,^13^. Some published papers theoretically and experimentally explore the extraordinary transmission occurring through electrically small diaphragms placed inside hollow pipe waveguides^14^,^15^,^16^,^17^,^18^,^19^. This work also considers this physical situation but adding ferrite elements to provide tunability. Aldin *et al*.^20^ obtained 740-fold transmission enhancement by exciting the electric resonance of SRR. In previous studies, our group obtained 300-fold transmitted enhancement by using Mie-resonance coupling of two ceramic resonators^21^. Based on Bethe’s theory^22^, light transmission through a single aperture of a radius *r* « *λ* scales with (*r*/*λ*)^4^. By using these configurations, the enhanced transmission which is more than that expected according to Bethe’s theory can be obtained.

Recently, to realize the miniaturization of the microwave system, tunable devices have got a great deal of attention^23^,^24^. Although many approaches on extraordinary transmission through a small aperture have been investigated, most of them can only realize the enhanced transmission at a certain frequency^25^,^26^,^27^,^28^. When the structure is determined, the frequency of the enhanced transmission can not be changed, which is not benefit for the development of miniaturization technologies.

Here, we report a magnetically tunable enhanced transmission by using two ferrite rods placed symmetrically on both sides of a single small aperture. With a certain applied magnetic field, a broadband transmission is obtained at the resonance frequency because the coupling effect of the ferromagnetic resonances of the two ferrite rods. Moreover, the resonance frequency of the enhanced transmission can be tuned by the applied magnetic field, which is benefit for the miniaturization of the microwave devices.

## Results

### Design of the extraordinary transmission through a small aperture

**Figure 1:**
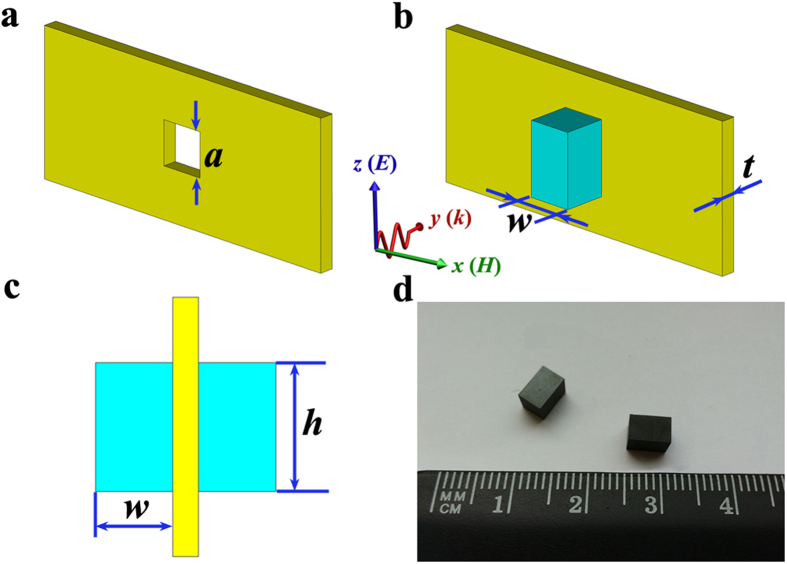
Schematic diagrams of a small aperture in a metallic plate and ferrite rods. (**a**) Copper plate with small aperture. (**b**) Perspective view and (**c**) cross-section (*yz*-plane) of the two ferrite rods symmetrically placed on both sides of the aperture. The electromagnetic wave (8–12 GHz) propagates along *y* direction with an electric field polarized along the *z* direction. (**d**) Photograph of the fabricated ferrite rods.

Figure 1a shows the schematic diagram of a single small square aperture with a size of 3 × 3 × 1 mm^3^ drilled at the center of a thin (*t* = 1 mm) copper plate. To realize ferromagnetic resonance coupling, two ferrite rods are placed symmetrically on both sides of the aperture, which is shown in Fig. 1b,c. The distance between the two ferrite rods is 1 mm. The electromagnetic wave (8–12 GHz) propagates along *y* direction with an electric field polarized along the *z* direction. Obviously, the length of the side of the square aperture is much smaller than the wavelength. Figure 1d shows the photograph of the fabricated ferrite rods.

Ferromagnetic resonance can arise in ferrite when a bias magnetic field is applied. The resonance frequency can be expressed as follows^29^:


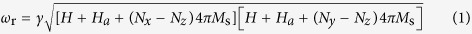


where *γ* is the gyromagnetic ratio, *H* is the applied magnetic field, *H*_a_ is the magnetocrystalline anisotropy field, *M*_s_ is the saturation magnetization caused by the applied magnetic field, *N*_x_, *N*_y_ and *N*_z_ are the demagnetization factor for *x*, *y* and *z* directions, respectively. According to Eq. (1), the two ferrite rods have the same resonance frequency when their material parameters (*H*_a_, *N* and *M*_s_) and *H* are the same. Moreover, the resonance frequency can be tuned by the *H*, which means that we can control the ferromagnetic resonance by adjusting the applied magnetic field and further we can obtain the magnetically tunable ferromagnetic resonance.

### Characterization of the proposed structure

**Figure 2:**
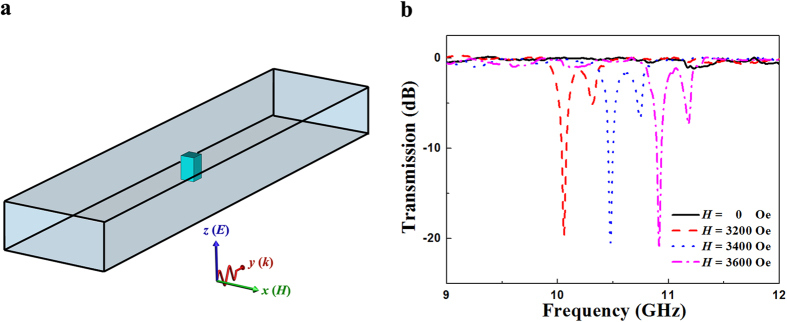
Experiment demonstrating the magneto-tunable behavior of the ferrite rod. (**a**) Schematic experimental setup of a ferrite rod placed in the rectangular waveguide. The applied magnetic field is along the *z* direction. (**b**) Measured transmission spectra for the ferrite rod under a series of *H*.

In order to confirm the magnetically tunable behavior of ferromagnetic resonance, the transmission spectra for the ferrite rod was measured. Figure 2a shows the schematic experimental setup of a ferrite rod placed in the rectangular waveguide. The applied magnetic field *H* is along the *z* direction. The measured transmission spectra for the ferrite rod under a series of *H* are shown in Fig. 2b. When *H* = 0, there is no significant dip in transmission spectra, which indicates that no resonance takes place. When a magnetic field of *H* = 3200 Oe is applied, a prominent transmission dip appears at 10.1 GHz, which is induced by the negative permeability of the ferrite around the ferromagnetic resonance frequency^30^. As *H* increases from 3200 Oe to 3600 Oe, the frequency of transmission dip increases, which exhibits a magnetically tunable behavior. In addition, a small transmission dip appears near the prominent dip, which can be attributed to the effect of the waveguide on ferrite rod.

**Figure 3:**
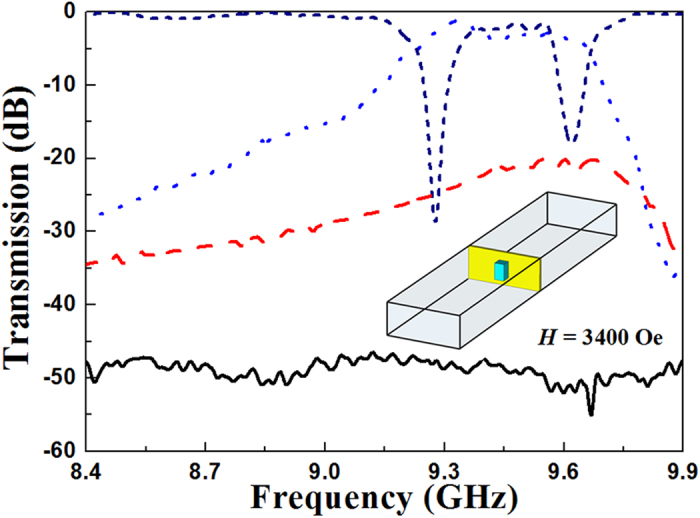
Experiment demonstrating broadband transmission through a single small aperture. The black solid line represents the measured transmission spectrum for the metallic plate without ferrite rods. The navy short dashed line represents the transmission spectrum when two ferrite rods are placed in the rectangular waveguide. The red dashed line represents the transmission spectrum when only one ferrite rod is coupled with the metallic plate. The blue dotted line represents the transmission spectrum for the metallic plate with two ferrite rods. The bias magnetic field of 3400 Oe is applied. The inset shows the schematic experimental setup.

Figure 3 shows the measured transmission spectra for a small aperture in a metallic plate with and without ferrite rods. The transmission spectrum for a small aperture in a metallic plate without ferrite rods is marked in black solid line. Because the length of the side of the square aperture is much smaller than the wavelength, the electromagnetic wave can not propagate through the metallic plate based on Bethe’s theory. According to wireless energy transfer principle^31^,^32^, two resonating objects with the same resonance frequency can exchange energy efficiently. Hence, by using the strong coupled ferromagnetic resonance, two ferrite rods with same material parameters were placed symmetrically on both sides of the aperture to realize the extraordinary transmission. The transmission spectrum for a small aperture in a metallic plate with ferrite rods is marked in blue dotted line. The inset shows the schematic experimental setup. The bias magnetic field of 3400 Oe is applied in the *z* direction. It can be seen that the transmission coefficients are above −2.5 dB over the frequency range from 9.25 to 9.6 GHz, indicating that a broadband transmission has been obtained. The maximum transmission coefficient is −0.98 db at 9.32 GHz, which demonstrates that nearly all of the electromagnetic energy has transmitted through the metallic plate. In addition, the transmission spectra when two ferrite rods are placed in the rectangular waveguide (marked in navy short dashed line) and when only one ferrite rod is coupled with the metallic plate (marked in red dashed line) are also shown in Fig. 3. Compared with the result shown in Fig. 2b, the ferromagnetic resonance when two ferrite rods are placed in the rectangular waveguide is stronger. It is obvious that the values of the spectrum for the metallic plate with two ferrite rods is much larger than that when only one ferrite rod is coupled with the metallic plate. Hence, the interaction between the two ferrite rods through the small aperture is much stronger than the other situations.

**Figure 4:**
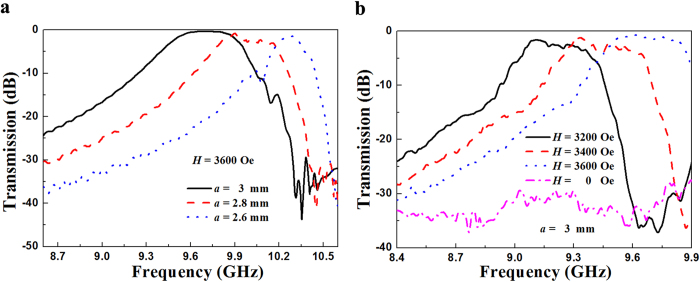
Experiment and simulation demonstrating magnetically tunable broadband transmission through a single small aperture. (**a**) Simulated transmission spectra for the small aperture with a series of side length *a*. (**b**) Measured transmission spectra for a small aperture in a metallic plate with ferrite rods under a series of *H*.

In order to understand the size effect of the aperture on the extraordinary transmission, the simulated transmission spectra for the small aperture with a series of side length *a* are shown in Fig. 4a. The bias magnetic field applied in the *z* direction is set at 3600 Oe. It can be seen that the frequency of transmission passband increases and the bandwidth becomes narrower as *a* decreases from 3 mm to 2.6 mm. To obtain the broadband property, the side length of 3 mm is chosen. Figure 4b shows the measured transmission spectra for a small aperture in a metallic plate with ferrite rods under a series of *H*. When *H* = 0, the values of the transmission coefficient are very low (about −33 db). Combined with the result shown in Fig. 2b, the reason is that there is no ferromagnetic resonance taking place. As *H* increases from 3200 Oe to 3600 Oe, the frequency of transmission passband increases, which indicates a magnetically tunable behavior. It can be seen that the tunable behavior observed here is in good agreement with that predicted in Eq. (1). The frequency of the transmission passband is inconsistent with that of ferromagnetic resonance shown in Fig. 2b, which is because the extraordinary transmission is induced by the interaction of ferrite rods and metallic plate perforated a small aperture. In addition, the maximum transmission coefficient increases from −0.98 db to −0.7 db, which demonstrates a nearly total transmission through a small aperture by ferromagnetic resonance coupling has been obtained. Note that this approach can not used in high frequency regions (THz, infrared or optical frequencies) due to the ferromagnetic resonance can not take place in these frequencies.

### Electric energy density distributions

**Figure 5:**
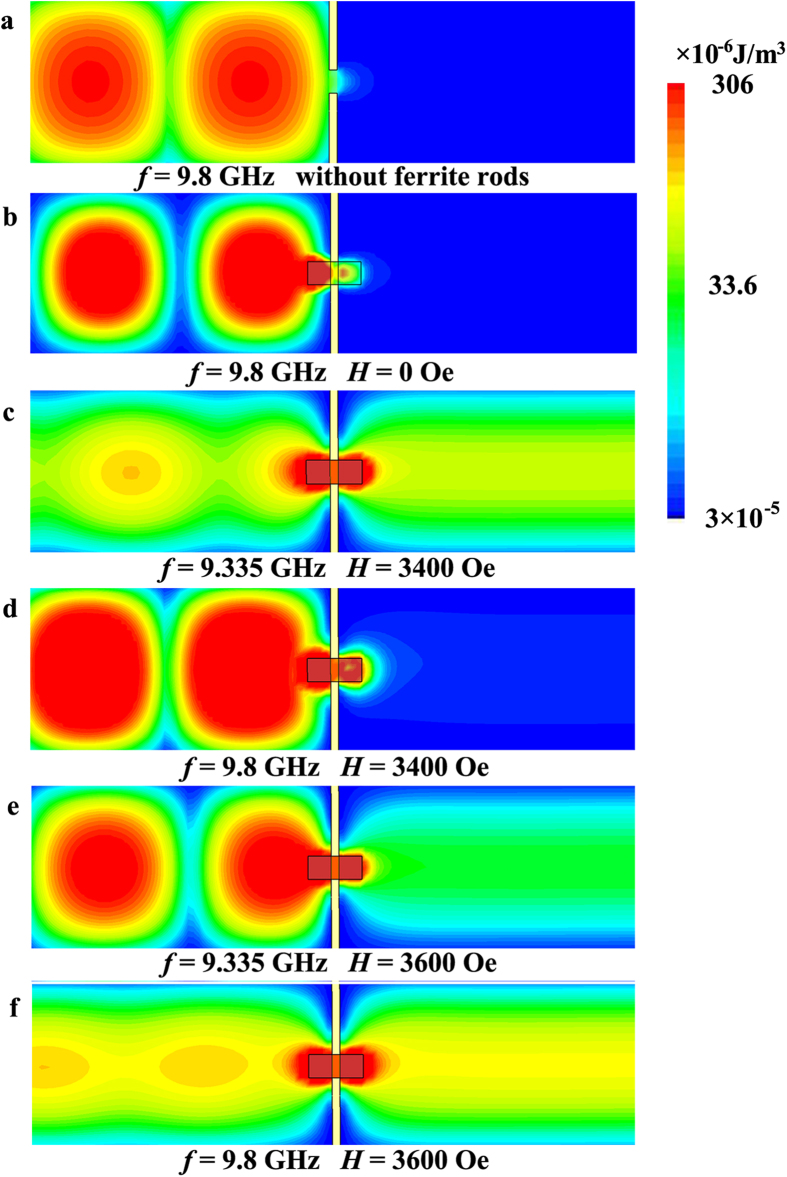
Electric energy density distributions showing the transmission characteristic. (**a**) Simulated electric energy density distribution in the *xy*-plane for a small aperture in a metallic plate without ferrite rods at 9.8 GHz. Simulated electric energy density distributions in the *xy*-plane for a small aperture in a metallic plate with ferrite rods under applied magnetic field (**b**) *H* = 0 Oe at 9.8 GHz, (**c**) *H* = 3400 Oe at 9.335 GHz, (**d**) *H* = 3400 Oe at 9.8 GHz, (**e**) *H* = 3600 Oe at 9.335 GHz and (**f**) *H* = 3600 Oe at 9.8 GHz.

To corroborate the above measured and simulated results, we have simulated the electric energy density distributions of various structures. Figure 5a presents the electric energy density distribution in the *xy*-plane for a small aperture in a metallic plate without ferrite rods at 9.8 GHz. The incident wave is coming from the left, shooting normally onto the metallic plate. There is no wave appeared in the left area of the plate because the length of the side of the square aperture is much smaller than the wavelength. Figure 5b presents the electric energy density distribution in the *xy*-plane for a small aperture in a metallic plate with ferrite rods under applied magnetic field *H* = 0 Oe at 9.8 GHz. Since the ferromagnetic resonance can not take place in the ferrite rods, there is also no wave appeared in the left area of the plate. Figure 5c,d show the electric energy density distributions in the *xy*-plane for a small aperture in a metallic plate with ferrite rods under applied magnetic field *H* = 3400 Oe at 9.335 GHz and 9.8 GHz, respectively. From Fig. 5c, it can be seen that the electromagnetic wave propagates through the small aperture. The ferrite rod resonator acts as an antenna which can efficiently receive (or transmit) electromagnetic energy in (or into) the waveguide. When a bias magnetic field of 3400 Oe is applied, the electromagnetic fields are extremely localized at the two ferrite rods, and the strongly coupling effect of the ferromagnetic resonances between the rods produces a nearly total transmission through the small aperture. At 9.8 GHz, little electric energy density appears in the left area of the plate (shown in Fig. 5d), which is in good agreement with the transmission coefficient of −24.8 db shown in Fig. 4b. Figure 5e,f show the electric energy density distributions in the *xy*-plane for a small aperture in a metallic plate with ferrite rods under applied magnetic field *H* = 3600 Oe at 9.335 GHz and 9.8 GHz, respectively. In the left area of the plate, the intensities of the electric energy density at 9.335 GHz and 9.8 GHz are in good agreement with the transmission coefficients of −10 db and −1 db shown in Fig. 4b. One observes that, as the *H* increases, the total transmission frequency increases. According to the above analysis, by using ferromagnetic resonance coupling between two ferrite rods, the magnetically tunable broadband transmission through a small aperture can be realized.

## Discussion

We numerically and experimentally demonstrated a magnetically tunable broadband transmission through a small aperture by ferromagnetic resonance coupling. Due to the strongly localized electromagnetic fields of the two ferrite rods and the efficient coupling effect, the maximum transmission coefficient reaches −0.7 db, which demonstrates a nearly total transmission through a small aperture has been obtained. More importantly, the total transmission passband can be tuned by the magnetic field. This work provides a way to realize magnetically tunable broadband transmission through a small aperture, which has greater potential for the microwave devices.

## Methods

### Sample fabrication

The ferrite rods were synthesized by using yttrium iron garnet (YIG) ferrite with relative permittivity *ε*_r_ = 14.5, saturation magnetization 4π*M*_s_ = 1950 Oe and linewidth Δ*H* = 10 Oe. The size of the YIG rods is 3 × 3 × 5 mm^3^. The metallic plate was cut to dimension of 22.86 × 10.16 × 1 mm^3^. The small square aperture with a size of 3 × 3 × 1 mm^3^ was drilled at the center of the copper plate. The schematic diagrams of the proposed structure and the photograph of the ferrite rods are shown in Fig. 1.

### Microwave measurements

The transmission spectra of the ferrite rod and the transmission through a small aperture with ferrite rods were measured by a microwave measurement system. The measurement system composed of a vector network analyzer (N5230C, Agilent Technologies, USA) and an electromagnet was shown in Ref. 33. The samples were placed in the X-band rectangular waveguides (22.86 × 10.16 mm^2^) and the waveguides were put in the middle of two magnets. By adjusted by input current, a bias magnetic field was generated around the samples along the *z* direction. The microwave propagated along *y* direction with the electric field along the *z* direction and the magnetic field along the *x* direction.

### Simulations

The commercial time-domain package CST Microwave Studio TM was used to carried out the numerical predictions of the transmission spectra and electric energy density distributions. All the parameters of the ferrite rods and the metallic plate were the same as those in the experiments. The relative permittivity, saturation magnetization and linewidth of the ferrite rods are set as 14.5, 1950 Oe and 10 Oe, respectively. The models in the simulations were set up in accord with the actual measurement environment. The boundaries of the rectangular waveguide in the *x* and *z* directions are set as perfect electric.

## Additional Information

**How to cite this article**: Bi, K. *et al*. Magnetically tunable broadband transmission through a single small aperture. *Sci. Rep*. **5**, 12489; doi: 10.1038/srep12489 (2015).
